# Supplementary material for: Hepatectomy or/with Metastatectomy for Recurrent Intrahepatic Cholangiocarcinoma: Of Promise for Selected Patients
Source: J Pers Med. 2022 Mar 29;12(4):540. doi: 10.3390/jpm12040540 (PMC9029635; doi:10.3390/jpm12040540)
Supplement: Supplementary file 1 [file jpm-12-00540-s001.zip › supplementary table S1.pdf]

Supplementary table S1 Clinicopathological characteristics of patients underwent hepatectomy for isolated intrahepatic recurrence

Gender/ Age	Extent of hepatectomy	Gross morphology	TNM stage	Recurrent site	Extent of repeat hepatectomy	Time to recurrence (months)	Survival after recurrence (months)
M/ 59	Seg 2,3 and partial Seg 5	MF	II	Right liver	partial Seg 4,5	10.1	1.5
M/ 69	Seg 2,3	MF	I	Left liver	Seg 4	103.1	2.2
M/ 79	Seg 2,3	MF	I	Left liver	partial Seg 4	10.3	10
M/ 66	Seg 6,7 and partial Seg 5,8	MF	I	Left liver	Seg 2,3	17.2	11.2
M/ 38	Seg 6 and partial Seg 5,7	MF	II	Right liver	Seg 8	11.9	11.8
M/ 70	Seg 5,6,7,8	MF	II	Left liver	Seg 4	10.7	13.4
M/ 43	Seg 7	MF	I	Left liver	partial Seg 3	46	28.6
M/ 67	Seg 1,2,3,4	mixed	IVA	Right liver	partial Seg 5	13.1	41
M/ 49	Seg 2,3,4	MF	I	Right liver	partial Seg 5,8	33.9	51.7
M/ 66	Seg 6,7	MF	II	Right liver	Seg 8 and partial Seg 5	32.4	61.6
M/ 59	partial Seg 4,5	MF	IVA	Right liver	Seg 6	14	159.6
F/ 63	partial Seg 2,3,4	MF	II	Left liver	Seg 3	93.8	174.2

Seg, segment of the liver; MF, mass forming type;
