# Supplementary material for: Hepatectomy or/with Metastatectomy for Recurrent Intrahepatic Cholangiocarcinoma: Of Promise for Selected Patients
Source: J Pers Med. 2022 Mar 29;12(4):540. doi: 10.3390/jpm12040540 (PMC9029635; doi:10.3390/jpm12040540)
Supplement: Supplementary file 1 [file jpm-12-00540-s001.zip › supplementary table S2.pdf]

Supplementary table S2 Clinicopathological characteristics of patients underwent surgery for locoregional recurrence

Gender/ Age	First hepatectomy	Gross morphology	TNM stage	Recurrent site	Extent of repeat operation	Time to recurrence (months)	Survival after recurrence (months)
F/ 41	Seg 5	MF	I	Regional lymph nodes	Hepatoduodenenal lymphadenectomy	17.6	88.7
F/ 40	Seg 2,3	PI	IVA	Omentum	Lesser omentum resection	9.0	16.0
F/ 47	Seg 5	MF	I	Diaphragm	Partial resection of diaphragm	34.0	40.0
F/ 43	Seg 2,3,4	IP	II	Cul de sac and ovary	Oophrectomy and hysterectomy	66.3	14.4
F/ 63	Seg 2,3,4	MF	I	Abdominal wall	Tumor excision	87.2	17.2
M/ 40	Seg 6	Mixed	IVA	Abdominal wall and omentum	Tumor excision	13.4	5.1
M/ 69	Seg 5,6	MF	I	Abdominal wall	Tumor excision	8.9	25.1
M/ 46	Seg 5	MF	III	Abdominal wall	Tumor excision	3.8	2.3
F/ 67	Seg 5	MF	II	Left liver and omentum	Seg 4 and tumor excision	15.7	4.6
M/ 53	Seg 1	MF	I	Left liver and peritoneum	Seg 4 and tumor excision	38.2	13.2
F/ 57	Seg 5,6,7,8	IP	I	Left liver and CBD	Partial Seg 4 and CBD resection	96.0	29.2

Seg, segment of the liver; MF, mass forming type; PI, periductal infiltration; IP, intraductal papillary; CBD, common bile duct
